# Popular Errors in Medicine

**Published:** 1893-07

**Authors:** James H. Dixon


// HALL'S

JOURNAL OF HEALTH

TRUTH DEMANDS NO SACRIFICE; ERROR CAN MAKE NONE.

VOL. XL. JULY, 1893. No. 7.

### POPULAR ERRORS IN MEDICINE.

A very common practice in eating such fruits as cherries or grapes is to swallow the stones, with the vague idea that these promote digestion. No error can be more fatally absurd. Many cases have occurred where such practices have been the cause of death, and that of a most excruciating nature. One instance is on record of a lady who died in great agony after years of suffering, and the cause was found to be several large balls found in the intestines accumulated around clusters of cherry stones. The husks of gooseberries are often swallowed with the idea that they prevent any bad effects from the fruit. On the contrary, they are the most indigestible substance that can be swallowed, and pass the stomach without any change, although they cause excessive irritation and not infrequently inflammation of the bowels.

Many people put great faith in the wholesomeness of eating only of one dish at dinner. They suppose that the mixture of substances prevents easy digestion. They would not eat fish and flesh, fowl and beef, animal food and vegetables. This seems a plausible notion, but daily practice shows its utter absurdity. What dinner sits easier on the stomach than a slice of roast or boiled mutton and carrots or turnips and the indispensable potato? What man ever felt the worse of a cut of cod or turbot, followed by a beefsteak or a slice of roast beef and pudding? In short, a variety of wholesome food does not seem incompatible at meals, *if one do not eat too much;* here the error lies.

It is a common practice with bathers, after having walked on a hot day to the seashore, to sit on the cold, damp rocks till they cool before going into the water. This is quite erroneous. Never go into the water if over fatigued, and after profuse and long continued perspiration, but always prefer plunging in while warm, strong and vigorous, and even with the first drops of perspiration on your brow.

There is no fear of sudden transitions from heat to cold being fatal. Many nations run from the hot bath and plunge naked into the snow. What is to be feared is sudden cold after exhaustion of the body, and while the animal powers are not sufficient to produce a reaction or recovery of the animal heat. There is a favorite fancy of rendering infants and farther advanced children, hardy and strong, by plunging them into cold water. This will certainly not prevent strong infants from growing stronger, but it will and often does kill three children out of every five. Infants always thrive best with moderate warmth and a milk-warm bath.

The same rule applies to the clothing of infants and children. No child should have so light clothing as to make it feel the effects of cold ;—warm materials, loose and wide-made clothing, and exercise are all indispensable for the health of little ones. But, above all things, their heads should be kept cool, and generally uncovered.

Many people so laud early rising as would lead one to suppose that sleep was one of those lazy, sluggish and bad practices, that the sooner the custom was abolished the better.

Sleep is as necessary to man as food, and as some do with one-third of the food that others absolutely require, so five hours sleep is amply sufficient for one, while another requires seven or eight hours. Some men cannot by any possibility sleep more than four or five hours in the twenty-four and therefore, true to the inherent selfishness of human nature, they abuse all who sleep longer.

No man should be taunted for sleeping eight hours if he can.

There is a common prejudice in the country that old women, farriers and professed bone-setters, are the only persons fit to prescribe for all sprains, dislocations and broken bones. Are these subjects less likely to be understood by an anatomist and regularly educated man, than the more difficult and intricate diseases which he daily treats with success ? What becomes of all such patients in large cities and hospitals, where a regular surgeon superintends their cure?

Have we so many stiff joints and deformed and useless limbs as among the patients of the empirical bone-setter ? Many people do not eat salt with their food and the fair sex have a notion that this substance darkens the complexion.

Salt seems essential for the health of every human being, more especially in moist climates such as ours. Without salt, the body becomes infected with intestinal worms. The case of a lady is mentioned in a medical journal, who had a natural antipathy to salt and never used it with her food; the consequence was, she became dreadfully infected with these animals. A punishment once existed in Holland, by which criminals were denied the use of salt; same consequence followed with these wretched beings. We rather think a prejudice exists with some of giving little or no salt to children. No practice can be more cruel or absurd.

One great cause of reluctance to medicine among the ignorant, is the idea, that many if not all of the powders and potions are made from human bones, and other parts of the body. In the present day no such thing exists; but yet nothing can better exemplify the saying "that popular prejudices are the cast-off clothes of philosophers, in which the rabble dress themselves," than the fact that even the great Lord Bacon believed in amulets; and Boyle seriously recommends the thigh-bone of an executed criminal as a powerful remedy in dysentery. Two-thirds of the medicine in common use are the dried roots, or leaves, or fruits, or gums of vegetables reduced to powder, or infused in water or spirit of wine; the other third are salts, obtained from sea water, from the waters of mineral springs, from burnt sea weed or land vegetables and from various preparations of the metals.

Many a child has turned with horror and disgust from a common emetic powder under the false conception that it was human liver pounded, when he would have even cheerfully taken off the nauseating draught had he been told that is was nothing more than the clean scraped roots of a beautiful little flowering plant that grows in warm countries, called ipecacuan. It is not an uncommon observation and sort of taunt, too, to the medical man that his drugs are all disagreeable to the palate. People do not reflect that this is a wise provision of nature. What, for instance, would be the consequence if the fruit whose pulp bears the bitter purging colocynth, were as inviting to the taste as a pine-apple?

Or how could the ignorant be restrained from every day poisoning themselves, if fox glove, hemlock or henbane bore sweet and enticing fruits? Another general reproach among the uninformed is, that inthe present day, physicians disdain to employ in their prescriptions the native plants of this country. This reproach is quite unfounded; there is never a day that some one of our native vegetables are not prescribed; but, undoubtedly, some of our most active and most valuable medicines can only be procured from hotter climates.—JAMES H. DIXON, M. D.

